# Domestic Summary

**Published:** 1840-04-11

**Authors:** 


### DOMESTIC SUMMARY.

### PHILADELPHIA DISPENSARY.

*Practical Obstetric Department.*

At a meeting of the class, held at the close of Dr. Warrington's Course, February 17th, 1840, Mr. J. H. Gamble of Virginia, Chairman, Mr. Thomas W. Harris of Tennessee, Secretary, the following preamble and resolutions were unanimously adopted:

While America may justly claim distinction in Schools of Practical Anatomy, Surgery, Clinical Medicine, Chemistry and Botany, it has hitherto been a source of regret, among the votaries of medical science, that no effective institution has, until recently, been established combining the practice of obstetrics with the theory; and that thus annually issued from the halls of her Universities, crowned with their highest honours, candidates for practice and fame comparatively unqualified for the duties of the Obstetrician.

*Resolved*, therefore, That we congratulate the rising members of the medical profession in general, and those in particular whose inclinations may lead them to the prosecution of obstetrics and female diseases exclusively, that the barrier hitherto existing to the attainment of this accomplishment is now entirely removed by the individual enterprise and untiring exertion of the Accoucheur of the Philadelphia Dispensary; who, while enlarging the sphere of his active benevolence to patients, adds to the facilities afforded by the institution over which he presides, a profound acquaintance with his subject, an established reputation, an extensive practice, unwearied zeal, and a felicity of imparting instruction peculiarly his own.

*Resolved*, That as the Practical Obstetric Institution of Philadelphia, is not only incomparably valuable in itself, in which the student renders practicable, in the lying-in-room, the theory of his preceptor, but as it is also the only one of the kind in the city, we take this opportunity of making the public aware of the advantages it affords.

*Resolved*, That as the close of the session unavoidably separates us from an institution so eminently interesting and instructive, we hereby testify our gratitude to Dr. Warrington as a benefactor, our admiration as a teacher, and our esteem as a gentleman.

*Resolved*, That the secretary wait upon the editors of the several medical journals of the city, requesting the publication of the foregoing preamble and resolutions.

*Resolved*, That Mr. S. Fuller of Pennsylvania, Mr. J. D. Mason of Tennessee, and Mr. Thomas W. Harris constitute a committee to transmit a copy of the proceedings of the meeting to Dr. Warrington.

J. H. Gamble, Chairman.

*Thomas W. Harris*, Secretary.

---

*Remarks on Hydrated Peroxide of Iron.* By William R. Fisher, M. D., late Professor of Chemistry and Pharmacy in the University of Maryland, &c. &c.—The peroxide of iron has recently acquired an importance in its therapeutical and pharmaceutical relations, from its use as an antidote against arsenious acid, which renders any apology for the notice here intended to be taken of it altogether unnecessary. Indeed, some notice of its properties as an antidote, mode of preparation, and preservation, seems to me to have become indispensable, from at least one erroneous view in regard to it, which appears still to prevail, and which may in some degree restrict its use and diminish its value. This error consists in the belief that the antidote, to be effectual, must be *freshly prepared*: a dictum which reached our shores simultaneously with the knowledge of the peculiar properties, as regards arsenious acid, of the oxide itself. Subsequently, each one who has written upon this subject has attached value to this italicised caution, and has thus perpetuated, at all events until now, a direction which has a tendency to banish or exclude a valuable remedy from the shops, and to compel the unfortunate victim of malice or accident to await the hurried preparation of the means by which his pangs may be alleviated. And this fresh preparation, too, has been required in the same breath, in which we are told that not a moment's time should be lost in the administration.

Having contributed in some measure to the continuance and extension of this belief, in a table of poisons and antidotes, prepared for the general therapeutics of Prof. Dunglison, published in 1836, it appears to be my particular duty to disabuse the pharmaceutic and medical public of this impression, and to show that the fresh preparation of the hydrate of peroxide of iron as an antidote to arsenious acid, is not necessary, and that any such view of its character is unsustained by sound philosophy or experience.

It is not necessary that the various authorities for the use and successful employment of this antidote should be here reviewed to establish its value. Enough has already been shown in other journals to satisfy the most incredulous, and it is only necessary to refer those who may yet need information on that score, to the contents of this Journal, vol. 10, page 263, and the American Journal of the Medical Sciences, vols. 15, 16, 20, 23, 24,* for experiments and cases which must prove fully satisfactory. Considering, then, its value as an antidote established by these records, let us examine the substance chemically, and see in what manner its character, composition, or properties can possibly be so altered by age or exposure, that its use would be rendered fruitless. It consists of iron combined with oxygen, and in those proportions, too, in which of all others these substances delight to combine. Into which combination, not only iron itself, but all its compounds containing less oxygen, spontaneously pass, when exposed to the air for any length of time; in other words, when kept. This state of oxidation, then, is that in which it is not difficult to retain the metal, and is one from which the metal does not spontaneously pass by exposure or prolonged keeping.

If it undergo no change from these causes, how can age affect it, or why must it be fresh? Will the carbonic acid of the atmosphere, by combining with it, neutralize its properties, and thus affect its value? Such is not the case. Peroxide of iron has less affinity for carbonic acid than the protoxide; so much so, that the precipitated carbonate of iron, which is a protosalt, when first formed, loses nearly

---

* Vol. 15, page 537, reported by Drs. Bunsen and Berthold.
" 16, " 239, " " Prof. Orfila and Dr. Leger.
" 20, " 222, " " Mr. John Robson.
" 23, " 503, " " Dr. John Murray.
" 24, " 243, " " Dr. Deville.

all its acid, by its base passing to the maximum of oxidation, as is well known, and established beyond a doubt. If kept with any kind of care, carbonic acid is the only acid to which the oxide could be exposed, and we have seen, that carbonic acid opposes no barrier to the preservation of the oxide. Theory, then, affords no reason why the antidote should be *freshly prepared.* Experience gives us an equally strong reason against it. In the latter part of September, or beginning of October, 1837, I was called upon in great haste for some *freshly prepared* antidote for arsenious acid, for a patient suffering from the poisonous effects of arsenic; with all possible expedition the antidote was prepared, but too late for the relief of the victim; although no time had been unnecessarily lost, the patient expired as the first doses were administered. During this preparation, which was the first I had made of this oxide, it occurred to me forcibly, that if it were suffered to remain diffused through the water in which it had been washed, that it would always be in the condition of a recent precipitate, and, in accordance with this view, it was so put aside. The antidote remained in the laboratory of the University of Maryland, unnoticed and untouched, except on one occasion, when it was exhibited to the medical class of the ensuing winter, as a specimen, until June 1st, 1838, when I was called upon by my friend, Dr. Thomas, for some of the antidote. Having none other to supply him with but this, prepared, at the least, eight months before, recourse was had to it. The result of its use is detailed by the doctor in the American Medical Library and Intelligencer, of July 16th, 1838. It is only necessary here to say, in regard to it, that the patient for whom it was employed, recovered.

The cases here quoted, serve the double purpose of showing the fatal consequences which may result from the loss of time consumed in the preparation of the antidote, and that *freshly prepared* hydrated peroxide of iron is not necessary to render arsenious acid insoluble and innocuous.

Theory and experience, then, both concur to sustain the position which I assumed at the commencement, and, I trust, will induce those whose province it is, to be constantly prepared with the means which have been shown to be worthy of firm reliance. Did not our knowledge of the properties of peroxide of iron teach us that it can undergo no change by age or exposure, I should not rely so confidently upon the single case known to me; but the result in that case was so exactly in conformity with the inductions from theory, that it deserves to be regarded as positive evidence.

Independently of my own observations, the experiments of Dr. Von Specz,* Professor of Chemistry in the Theresian Academy of Vienna, sustain the opinion herein advocated, by showing, 1st, "that this preparation, when properly made, and preserved in bottles, with good glass stoppers, will retain its virtues for a very considerable time." 2d, "that rust of iron, and hæmatite, although they do not prevent all the bad effects of arsenic on the system, may, in defect of the hydrated peroxide of iron, be employed as antidotes to that poison." And the case reported by Mr. John Robson* also corroborates this view. He having no hydrated peroxide of iron at hand, administered six drachms of the carbonate of iron in water, in two draughts. "The patient said his stomach felt cooler. His pulse fell from 130 to 112. The pain ceased, or nearly so." Now it will be observed that in none of the cases here referred to, was *freshly prepared* hydrated peroxide of iron the agent by which the antidotal effects were produced. Dr. Von Specz employs the powder in all his experiments with the hydrated oxide, which could not have been an immediate preparation, finds that it can be preserved a considerable time, and finally discovers that other peroxides may be used in default of the hydrate, one of which is as old as the creation, the other, age unknown. It is true the hydrate is always to be preferred, but its place can be supplied. Dr. Robson also gave the hydrated oxide as soon as it could be got ready, (an hour and a half after the carbonate was given,) but his patient had been already relieved by the *old* preparation, first swallowed. The recent preparation was not *well washed*, and many urgent symptoms supervened upon its use. "He (the patient) said he felt sick, and worse after taking the physic." The next morning, the prepared oxide, more carefully washed, was given. "It was not so good to take, but he had no more sickness," &c.

These, then, it is asserted, prove that age is no obstacle to the effects of peroxide of iron; that it can be kept, and that the precipitated peroxide must be thoroughly washed. It can scarcely be necessary to remind the readers of a scientific journal that the carbonate of iron, and hæmatite, are almost wholly peroxide of iron. The former containing a trifling amount of carbonic acid; the latter, perhaps, some siliceous or earthy matter. My own view is, that these would be equally efficacious as antidotes, if in an equally impalpable state, with the precipitated peroxide.

The only possible reason which I can conceive for requiring the oxide to be freshly prepared, is that it may be administered in as finely divided a condition, as nearly approaching solution as possible; and this certainly can only be accomplished by employing it in the pulpy state of a recent precipitate. This state

* American Journal of Medical Sciences, Vol. 21, page 519.

* American Journal of Medical Sciences, Vol. 20, page 222.

is, however, not inconsistent with age, and may always be preserved, for any reasonable length of time, as I know by experiment, simply by suffering enough of the water with which the precipitate has been washed, to remain, in order that the oxide may be diffused through it. So very minutely divided is the oxide in its precipitated state, that a trifling agitation serves to distribute it promptly through the supernatant fluid, which thus forms a medium for its administration, as well as a means for apportioning the dose. This mode is that which has been employed in the preservation of all the hydrated peroxide which I have ever made. Theoretical considerations induced me to employ it, counter to the dictum against which I now write; and experience, in the case already quoted, so fully satisfied me of the advantages to be derived from it, that I at once commenced the preparation of a large supply of the antidote, so that any future case might not be deprived of the benefit which can be obtained from its immediate use. Some of the oxide, prepared in June, 1838, is now by me, and to all appearance is entirely unchanged. No critical examination could distinguish it from a preparation a week old. Several friends, who have seen it, concur in this opinion.

It was my intention to have here rested the argument, but just as it was completed, I was indebted to the kindness of Mr. Durand, who, ever anxious for the diffusion of information, and the improvement of our science, placed in my hands the twenty-fourth volume of the "Journal de Pharmacie," Paris, 1838, containing a communication from Drs. Bunsen and Berthold, "*On the mode of preparing in the most convenient form the Hydrated Sesquioxide of Iron, as an Antidote to Arsenious Acid.*" To these gentlemen we owe this employment of the hydrated oxide, and I therefore, with the greater pleasure, adduce their testimony in favour of the views which have been herein urged.

As the subject now under discussion is in regard to the preservation of the oxide, the latter portion of their paper is first quoted, in their own words: "It is altogether inconceivable," say they, "that any one, relying upon uncertain experiments with animals, should have recommended the preservation and use of the antidote in a dry state, since a number of experiments already made, coinciding with our own, tend to this result, that the action of the sesquioxide of iron is null, and that of the *dry* hydrate incomplete; of which, the simple fact that the dry hydrate does not ever precipitate, wholly, in the cold arsenious acid, should have previously apprised every one."

The conclusions, then, which I consider established, both by the evidence here offered, and the absence of evidence to the contrary, are

1st. That we are justified in believing that peroxide of iron undergoes no change from age, by inferences drawn from the known great affinity of iron for oxygen.

2d. That the discovery of native peroxide, entirely unchanged, affords positive testimony of the foregoing position.

3d. That relief has been afforded by the use of the peroxide of indeterminate age.

From which, as a necessary consequence, it follows that neither reason nor observation sustains the opinion, that the precipitated hydrate of peroxide of iron *must be freshly prepared* to render it available as an antidote to arsenious acid.

In regard to the precipitated hydrate of peroxide of iron, it is proved,

1st. That, owing to its state of aggregation, when moist, it is the preferable form in which to administer it as an antidote.

2d. That it should be well washed before being so employed.

3d. That the extemporaneous preparation of the antidote is inadvisible, because time is lost in the administration, beside the inability to wash it.

4th. That the hydrated peroxide of iron can be preserved any length of time unaltered, and ready for immediate use by suffering it to remain diffused through a portion of the water in which it has been washed, corked up in bottles.*

And lastly, That every apothecary, and physician residing in the country, should always be provided with the antidote thus preserved in bottles of a convenient size for use.

Enough, has now, certainly been said to establish the position which I undertook to demonstrate, and a few comments upon the mode of preparation are now offered. Several modes have been suggested for the preparation of this oxide, which, although varied in detail, are essentially the same in principle: effecting the oxidation of the iron to its maximum extent, by the decomposition of nitric acid, and precipitating the oxide thus obtained by ammonia.

It is unnecessary to review these formulæ in detail, further than to indicate a few reasons for the preference given to the formula here proposed. If nitric or nitro-muriatic acid be employed as the solvent of iron, as prepared by one author, violent and inconvenient action, and great heat, attend the operation, and the clothing and utensils of the operator encounter some risk. To this objection our formula is not exposed. And the direction given by the same authority for "*drying the powder in the shade*," is entirely inconsistent with our

* These first, second, and fourth positions receive additional strength from the directions given by Drs. Bunsen and Berthold for making this oxide. They will be mentioned further on, and although not italicized in the text, have been so printed here to distinguish them.

whole object, the preservation of the oxide in a pulpy state. Another writer, anonymous, it is true, but one who evidently understands his subject, offers two formulæ for this preparation. The former of which, with the exception of not affixing quantities, coincides exactly with our mode of preparation, although nothing is said by him of preservation, and one expression of which is quoted in his own words, because reference will shortly be made to it: "The alkali throws down the hydrated peroxide as a reddish precipitate, which must be carefully washed." In his second process, he speaks of its extemporaneous preparation, "by boiling aqua fortis in a common iron pot, with some iron filings or nails, for a few minutes, pouring off the clear liquor, and then adding to the fluid a saturated solution of carbonate of soda, the hydrated peroxide will be precipitated in the form of a reddish powder. A saturated solution of the nitrate of iron, as also of the carbonate of soda, may be kept in separate bottles in the office of a physician, and the antidote made whenever required, by merely mixing a portion of each solution with the other." To this formula and recommendation there are two objections, of which one may be regarded as serious, viz.: that by this extemporaneous preparation, the oxide is not washed, and the highly irritated, if not inflamed, mucous coats of the stomach and œsophagus are deluged with a concentrated solution of nitrate of soda, "whose irritant properties," says Professor Ducatel, in his Abridgement of Christison, "will be found, most probably, to produce the same effects on the animal system as the nitrate of potassa." Such analogy should certainly deter us from the use of the extemporaneous preparation. Indeed, the author himself says above in his first formula, the precipitate "must be carefully washed," indicating his own views of the necessity of removing the new alkaline salt, and exhibiting a decided inconsistency between his formulæ, which nothing but an accidental oversight could have caused.* The other objection to this formula is of less importance and has already been suggested in the first comment made upon the use of nitric acid for dissolving the iron.

It is believed that no objection of moment can be made to the proposed formula, herein commended; indeed, the chief defects in those already reviewed, arise from the anxiety to employ the preparation in its recent state, which it has been the object of this notice to prove unimportant. That it did in one case at least deprive the sufferer of its benefits, and, consequently, of his life, I am fully persuaded; and seriously have I regretted having given implicit credence, without proper reflection, and a recurrence to principle, to the idea that only a freshly prepared oxide could be relied upon.

The formula now offered is definite in its proportions, and if carefully observed, will furnish a result upon which reliance may be placed. It is based upon the equivalent proportionals of the materials, and is made to coincide as nearly as possible with the preparations of the United States Pharmacopœia, the officinal acids being employed. At my request, it has been subjected to practice by Mr. Durand, who has obtained a very perfect result, with a satisfactory economy of material.

*Hydrated Peroxide of Iron.*

| | | | |
|---|---|---|---|
| ℞. Sulphuric Acid, | (67° Baumé,) | 8 oz. | 16 pts. |
| Iron Wire, | | 8 oz. | 16 " |
| Nitric Acid, | (49° Baumé,) | $5\frac{1}{2}$ oz. | 11 " |
| Water of Ammonia, | | q. s. | |
| Water, | | $1\frac{1}{2}$ gal. | 384 " |

Mix the sulphuric acid with the water in a glass vessel. Add the iron, and, after the effervescence has ceased, filter. Add the nitric acid in divided portions, and apply heat so long as orange coloured fumes are given off. To the heated solution, pour in the water of ammonia until a decided excess has been added, then wash the precipitate by decantation, until the washings give no precipitate with nitrate of baryta. The water is then to be drawn off until just enough remains to give the consistence of thick cream. It should be introduced into bottles of convenient size for use.

Bottles containing half a pint are recommended as convenient; and the annexed direction, it is thought, will enable the most ignorant to use it until medical advice can be obtained. "This antidote must be administered *as soon as possible* after the discovery that arsenic has been taken, and as it produces no bad effects itself, should be given *every five or ten minutes, until entire relief is obtained.** The dose for a grown person is a table-spoonful; for children, a dessert-spoonful. The bottle must be well shaken before each dose."

The following remarks on the preparation are from the paper of Drs. Bunsen and Berthold. After giving directions for the preparation, by means of sulphuric acid, iron, nitric acid, and ammonia, without specifying propor-

* It may appear hypercritical to allude to the irritant properties of these nitrates, when so powerful an irritant as arsenic is to be counteracted, and if their presence was unavoidable, the comment might be attributed to an ultra disposition to find fault; but, as it has been shown that the properties of the antidote may be enjoyed unattended with accompanying irritation, these remarks are made with an honesty of purpose which, it is trusted, will be ample to disarm them of any apparent malice or discourtesy to the author.

* It is considered better to administer it thus in doses until relief is obtained, than to endeavour to give four, eight, or twelve times the amount of arsenic taken, which, for obvious reasons, can seldom be known.

tions, they proceed: "It is necessary not to lose sight of the fact, that the solution of the salt of iron must be complete before adding the nitric acid in small quantities, otherwise, a considerable amount of a neutral sulphate of peroxide will separate in the form of a yellowish powder, which is very slightly soluble.

The chloride of iron affords a means of preparing this body still less eligible, because the risk is run in precipitating, by ammonia, of obtaining an admixture of a large quantity of subchloride of iron."

"*In order that the hydrated sesquioxide may not be deprived of its water, and by this means of diminishing, in the least possible degree, its feeble state of aggregation, it should not be filtered, but after having been suffered to subside for several days, the supernatant fluid being poured off, it must be preserved under water in closed vessels.*"

"Simple as is the process here indicated for the preparation of the antidote, there have been, nevertheless, modifications proposed, some of them so unfit that we believe it useful to add some remarks on this subject. First of all, there is one practice which ought to be rejected, that of employing another alkali than ammonia for the precipitation of the hydrate of the sesquioxide of iron, as some have done; in fact, the least quantity of alkali retained in the precipitate will give rise to the formation of an arsenite, which would abstract itself entirely beyond precipitation by the hydrate of sesquioxide of iron, because, although this base can prevail over the affinity of ammonia for arsenious acid, it could not over that of soda or potassa."*

The subject is now submitted for the deliberate examination of the two professions who are interested in its determination, and upon whom no greater reward can be bestowed for the labour of the investigation which it merits, than the reflection that they are about to increase their ability for usefulness, and to divest the dreadful anticipation of poison of some of its risks and horrors. In treating this important question, my sole motive has been the promulgation of truth, and banishment of error; and in combatting the opinions of many who are entitled to the highest respect and confidence, I have deemed it indispensable to my own immunity from a charge of rashness, to produce evidence of the strongest character, depending upon facts which, it is believed, cannot be refuted. The introduction of therapeutical considerations has been avoided as much as possible, and only employed when essential to establish the value of particular preparations, and to enlighten pharmaceutical research.

*Philadelphia, March* 27, 1840.

* This may have been the mode employed by Dr. Robson, whose patient found himself worse after having been relieved, upon using an imperfectly washed precipitate; and felt no pain after using some which had been well washed. See Amer. Journ., Vol. 20, page 222.

